# Potential mechanisms and modifications of dietary antioxidants on the associations between co-exposure to plastic additives and diabetes

**DOI:** 10.1038/s41387-024-00330-1

**Published:** 2024-09-03

**Authors:** Yang Yang, Cheng Zhang, Hui Gao

**Affiliations:** 1 Department of Prevention and Health Care, Affiliated Jinhua Hospital, Zhejiang University School of Medicine, Jinhua, 321000 Zhejiang China; 2 Department of Oncology, The First Affiliated Hospital of Anhui Medical University, No.218 Jixi Road, Hefei, 230022 Anhui China; 3 Department of Biostatistics, Anhui Provincial Cancer Institute, No.218 Jixi Road, Hefei, 230022 Anhui China; 4 Department of Pediatrics, the First Affiliated Hospital of Anhui Medical University, No.218 Jixi Road, Hefei, 230022 Anhui China

**Keywords:** Risk factors, Nutrition, Epidemiology

## Abstract

**Background:**

The association of plastic additive mixture exposure with diabetes and the modifying effects of dietary antioxidants are unclear.

**Methods:**

The data from the NHANES 2011–2018 were retrieved, and phthalates and organophosphate esters (OPEs) were selected as exposures. The coexposure effect was analyzed by the environmental risk score (ERS) and quantile g-computation. To mitigate any potential bias caused by using the internal weights, another version of ERS was constructed using the cross-validation approach. The level of dietary antioxidant intake was measured by the composite dietary antioxidant index (CDAI). The biological mechanism underlying the association was studied by the adverse outcome pathway (AOP) framework.

**Results:**

Fifteen chemicals (ten phthalates and five OPEs) were measured in 2824 adult participants. A higher ERS was significantly associated with an increased risk of diabetes (OR per 1-SD increment of ERS: 1.25, 95% CI: 1.13–1.39). This association apparently interacted with the CDAI level (OR_low_: 1.83, 95% CI: 1.37–2.55; OR_high_: 1.28, 95% CI: 1.15–1.45; *P*_interaction_ = 0.038). Moreover, quantile g-computation also revealed higher level of combined exposure was positively associated with diabetes (OR: 1.27, 95% CI: 1.05–2.87), and the addition of dietary antioxidants showed a null association (OR: 1.09, 95% CI: 0.85–2.34). The AOP study identified TCPP and TCEP as key chemicals that cause aberrant glucose metabolism and insulin signaling pathways and result in diabetes.

**Conclusions:**

Coexposure to phthalates and OPEs is positively associated with diabetes, where an antioxidative diet plays a modifying role. Several potential mechanisms have been proposed by AOP framework.

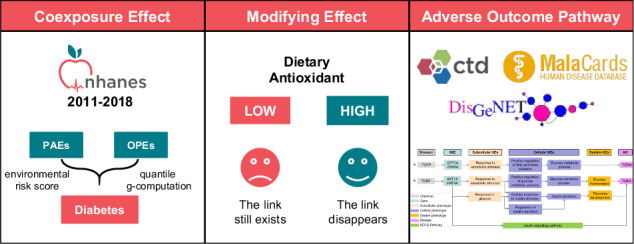

## Introduction

Plastic materials are indispensable durable materials in modern daily life. They are widely used in various fields, such as automobile, agricultural, healthcare, construction, toy, packaging, and textile production [1]. Annual global use reached 460 million metric tons in 2019, and this value is expected to nearly triple by 2060. Low-cost and durable plastic products are convenient for use in our lives. However, a significant amount of plastic waste is generated each year, accounting for 54% of the global anthropogenic waste quality [2]. The large production volume, low recycling rate, and insufficient sustainable policies supporting plastic recycling have led to a large influx of plastic waste into the environment. Plastic waste is toxic when it contacts different species, including humans. Toxicity can be caused by the polymer matrix, additives, degradation products, and adsorbed pollutants [3]. According to previous reports, polyvinyl chloride is the most toxic type of polymer (monomer or additive) commonly used in daily life; additionally, additives are more toxic to wildlife and humans than monomers [1].

Presently, the most common chemical additives used, such as flame retardants, and plasticizers, such as phosphate-based flame retardants and phthalate esters (PAEs), pose significant threats to human health [3]. PAEs are currently the most widely used plasticizers and one of the most toxic additives in polyvinyl chloride products [1]. The global annual production of PAEs can reach 11 billion pounds. Organic phosphate esters (OPEs) are widely used as substitutes for brominated flame retardants in production and daily life. The global consumption of OPEs was 500,000 tons in 2011, and this number increased to 680,000 tons in 2015 [4]. In addition to being a flame retardant, OPE is also used as a plasticizer and solvent and in other industrial applications [5]. PAEs and OPEs are used as plasticizers, flame retardants, solvents, and in other industrial applications [5, 6]. These additives are not chemically bound to the products and are vulnerable to volatilization and leaching into the environment. The combined exposure levels of PAEs and OPEs are greater in environmental media and in living organisms, including humans [7–10]. Ingestion, inhalation, and skin contact are the main pathways of coexposure to PAEs and OPEs. Studies have shown that coexposure to PAEs and OPFRs is associated with adverse early reproductive outcomes resulting from in vitro fertilization [11], childhood asthma resulting from disruption of inflammatory lipids and fatty acid metabolism [10], and neurodevelopmental deficits [12]. Although a positive correlation between PAE or OPEs alone and the risk of diabetes in adults has been found [13–15], the effect of their combined exposure on diabetes has not been studied.

However, the biological mechanism underlying the impact of PAEs or OPEs exposure on the risk of diabetes is still unclear. The adverse outcome pathway (AOP) is a framework commonly used in the field of computational toxicology to explore the toxic action patterns of chemicals. By integrating public database resources, such as the comparative toxicogenomics database (CTD), disease gene network (DisGeNET), and MalaCards human disease database, researchers have effectively developed some AOPs for potential mechanistic pathways between chemical exposure and disease [16–19].

Interestingly, evidence has suggested that PAE exposure is associated with increased oxidative stress, which mediates the development of insulin resistance and diabetes [20, 21]. The positive association between PAE exposure and insulin resistance weakened among participants with higher concentrations of serum β-carotene, which is an abundant antioxidant [22]. In addition to β-carotenoids, a large amount of other vitamins and trace elements in food are antioxidants. Studying the protective effects of antioxidant nutrient intake on the health hazards of PAE and OPE exposure is highly important.

Utilizing NHANES data from 2011 to 2018, this study aimed to: (1) analyze the association between exposure to key substances of PAEs and OPEs combined and diabetes; (2) investigate whether antioxidant diets can modify this association; and (3) identify the potential biological mechanism behind the increased diabetes risk associated with PAEs and OPEs.

## Methods

### Study design and population

The NHANES is an ongoing national cross-sectional survey specifically designed to evaluate the health and nutritional status of representative individuals of the U.S. civilian noninstitutionalized resident population. Recognizing that the measurement of PAE and OPE in subsamples might not yield sufficient stability for robust analyses within a single survey cycle, we extracted and combined data from four survey cycles spanning the years 2011–2018, which included 5434 participants with information on the concentrations of PAEs and OPEs, self-reported diabetes and other covariates. After excluding individuals who were younger than 18 years (*n* = 2046), pregnant (*n* = 29), or missing data for the outcome (*n* = 3) or for exposure and other covariates (*n* = 514), a total of 2824 individuals were included in the analysis (Fig. S1). Diabetes status was determined through the question “Did you have diabetes or sugar diabetes?” The respondents who refused to answer or did not know the answer were treated as missing. Since some participants had borderline hyperglycemia, we created two scenarios of diabetes: the first scenario included patients with diabetes and borderline high blood sugar levels, while the second scenario did not include the borderline levels. We conducted the primary analyses with the outcome defined as diabetes in the first scenario and then repeated the procedure by changing the outcome to diabetes in the second scenario.

### Measurement of PAEs and OPEs in urine

The concentrations of PAEs and OPEs in urine were measured in participants aged 6 years and older from a one-third subsample by NHANES researchers using analytical methods that have been previously described elsewhere [23, 24]. Briefly, the method uses 0.2 mL of urine and is based on enzymatic hydrolysis of urinary conjugates of the target analytes, automated off-line solid-phase extraction, reversed-phase high-performance liquid chromatography separation, and isotope dilution-electrospray ionization-tandem mass spectrometry detection. For this study, we focused on ten PAEs and five OPEs with missing data rates lower than 30% [25] (details in Table S1), including MEP (mono-ethyl phthalate), MnBP (mono-*n*-butyl phthalate), MiBP (mono-isobutyl phthalate), MBzP (monobenzyl phthalate), MEHHP (mono-(2-ethyl-5-hydroxyhexyl) phthalate), MEOHP (mono-(2-ethyl-5-oxohexyl) phthalate), MECPP (mono-2-ethyl-5-carboxypentyl phthalate), MCPP (mono-(3-carboxypropyl) phthalate), MCNP (mono (carboxynonyl) phthalate), MCOP (mono (carboxyoctyl) phthalate), BCPP (bis(1-chloro-2-propyl) phosphate), BCEP (bis(2-chloroethyl) phosphate), BDCP (bis-p-cresyl phosphate), DBUP (dibutyl phosphate), and DPHP (diphenyl phosphate (DPHP). In cases where the result was below the limit of detection, the value for that variable was the detection limit divided by the square root of 2. To mitigate the impact of diluted urine, PAEs and OPEs concentrations were adjusted for urinary creatinine and were expressed as μg/g creatinine.

### Covariates

The selection of covariates for this analysis was based on previous knowledge about the association between environmental exposure and diabetes. The included covariates were time of sampling, sex, age, race, marital status, education, body mass index (BMI), household poverty-to-income ratio, smoking status, alcohol consumption status, hypertension status, gout status, family history of diabetes, daily intake of nutrients (total energy, fat, protein, and carbohydrate), alanine transaminase (ALT), aspartate aminotransferase (AST), blood urea nitrogen (BUN), and creatinine.

We used the composite dietary antioxidant index (CDAI) to describe the level of antioxidants in the diet. The CDAI was calculated by adding the six normalized vitamins and minerals, including vitamins A, C, and E, selenium, zinc, and carotenoids from food only [26]. The diet-derived intake information was obtained from a detailed dietary interview component that estimated the types and amounts of foods and beverages consumed during the 24-h period prior to the interview. The CDAI score was subsequently transformed into a binary variable (low/high) according to the 75th percentile value.

### Statistical analysis

All analyses were performed using R software (Vienna, Austria, version 4.3.0), and a *P* value less than 0.05 was considered as significant. The demographic characteristics accounting for the sampling design effect were shown as the median (p25, p75) and percentage (standard error) for continuous and categorical variables, respectively. To test the equality of two medians or two proportions, the design-based rank test or Rao‐Scott chi‐square test was used. The exposure levels of each chemical were shown by weighted percentiles (5th, 25th, 50th, 75th, and 95th). The Spearman correlation coefficients of each pair of chemicals were calculated and illustrated by a heatmap. These procedures were implemented using the “survey” package [27].

To investigate the association between single exposure (logarithm-transformed) and diabetes, survey-weighted logistic regression was used, and odds ratios (ORs) with 95% confidence intervals (CIs) were calculated. The strategy of covariate adjustment was as follows: Model 1 included sampling time, age, sex, poverty status, race, education, marital status, smoking status, drinking status, BMI, hypertension status, gout status, and family history of diabetes; Model 2 additionally included total nutrient intake, liver function, and kidney function. These procedures were implemented using the “survey” package [27].

We computed the environmental risk score (ERS) to assess the cumulative risk of diabetes from exposure to PAEs and OPEs [28]. The score is a weighted sum of exposed chemicals, whose weights (β) are derived from the adaptive elastic net (adENET) method. This method utilizes two tuning parameters (lambda 1 and lambda 2) to select the subsets of chemicals within the mixture that are most predictive of individual diabetes and to quantify the relationship between pollutants and diabetes. Specifically, lambda 1 shrinks the coefficients of unimportant predictors exactly to zero, and lambda 2 stabilizes selection in the presence of highly correlated predictors. Five-fold cross-validation and optimization of cross-validated prediction errors were used to estimate lambda 1 and lambda 2 (Fig. S2a). To avoid overfitting issues and mitigate any potential bias caused by using the internal weights, we constructed another ERS (ERS_CV) by a cross-validation approach as a supplementary analysis. Briefly, the data were randomly divided into 5 folds, with weights for each fold predicted from the adENET model trained on the other fourfolds [29]. To evaluate the effect of mixed exposure, scaled ERS and the tertile category of ERS were used in the logistic regression adjusting for Model 2 covariates. The linear trend across tertiles of ERS was additionally tested by assigning 1–3 for the tertile and modeling this value as a continuous variable. These procedures were implemented using the R packages “gcdnet” [30] and “caret” [31].

To ensure the robustness of the findings, a quantile g computation (qg-computation) model was also used to evaluate the combined effect of PAEs and OPEs on diabetes, conditional on no covariate (crude model) or Model 2 covariate (adjusted model). The overall mixture effect from the qg-computation model (psi1) is interpreted as the effect on the outcome of increasing every exposure by one quantile. These analyses were implemented using the “qgcomp” package [32].

The modifying effect of the CDAI on the association between mixed exposure and diabetes was examined by adding an interaction term (ERS_scaled_ × CDAI_low/high_) to the weighted logistic regression model and testing whether the *P* value for the interaction was significant. In addition, the modifying effect was investigated by adding CDAI components to the aforementioned qg-computation model. We hypothesized that a modifying effect would occur if the mixture effect of the new model reversed to null, which implied that adding antioxidant nutrients would decrease the positive mixture effect of PAEs and OPEs.

### AOP development based on public databases

The research results suggest that the metabolites TCEP and TCPP have the highest weights under additive mixture exposure. Type 2 diabetes mellitus (T2DM) is the main type of diabetes in adults and is considered an “adverse outcome (AO)”. On January 14, 2024, the “Gene” and “Phenotype” tags of TCEP and TCPP were searched in the CTD database. The TCEP- and TCPP-related genes and phenotypes were obtained and are referred to as “TCEP-gene”, “TCPP-gene”, “TCEP-phenotype”, and “TCPP-phenotype”, respectively. In addition, under the “disease” tag in the CTD database, candidate genes for T2DM associated with TCEP and TCPP were identified and named “TCEP-gene-T2DM” and “TCPP-gene-T2DM”, respectively. Similarly, information related to the T2DM gene and T2DM phenotype was obtained from the DisGeNET and MalaCards public databases. A summary of the collected genes and phenotypes is shown in Table S2.

**Fig. 1:**
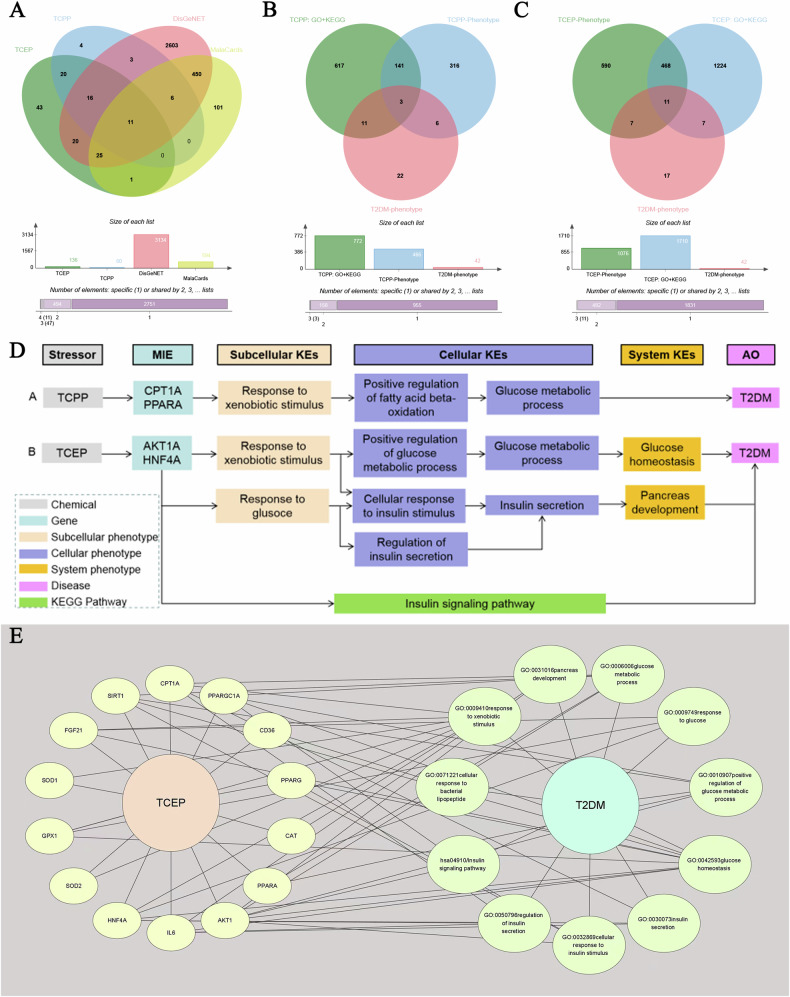
Construction of AOP from TCPP or TCEP to T2DM. **A** Genes derived from the CTD, DisGeNET, and MalaCards. **B** Target phenotypes derived from GO and KEGG pathway enrichment analyses, CTD, and MalaCards for TCPP. **C** Target phenotypes derived from GO and KEGG pathway enrichment analyses, CTD, and MalaCards for TCEP. **D** AOP networks from TCPP or TCEP to T2DM. **E** TCEP-Gene-Phenotype-T2DM network. CTD comparative toxicogenomics database, DisGeNET disease gene network, GO gene ontology, KEGG Kyoto encyclopedia of genes and genomes, AOP adverse outcome pathway, T2DM type 2 diabetes mellitus, TCEP tris(2-chloroethyl) phosphate, TCPP tris(1-chloro-2-propyl) phosphate.

A Venn diagram was generated to identify the genes intersecting the TCEP-gene or TCPP-gene with the T2DM gene (Fig. 1A). The genes were subsequently subjected to Gene Ontology (GO) and Kyoto Encyclopedia of Genes and Genomes (KEGG) enrichment analyses using the package “clusterProfiler” [33]. These analyses focused on *Homo sapiens* as the source organism. Furthermore, these statistically significant GO and KEGG analysis results (*P* < 0.05) were intersected with the T2DM phenotype to obtain the target phenotype set (Fig. 1B). To obtain more reliable genes related to T2DM and chemical exposure, the above genes were merged with the TCEP-gene-T2DM or TCPP-gene-T2DM gene sets to obtain the two target gene sets. Finally, the interaction network between the target gene set and target phenotype set was visualized using Cytoscape 3.8.0 (https://cytoscape.org/) if available. On the basis of this information, a putative AOP was proposed, serving as a potential clue for understanding the mechanism through which TCPP/TCEP may contribute to the development of T2DM.

## Results

### Demographic characteristics

The median age and BMI of the overall participants were 48 years and 28.2 kg/m^2^, respectively, with interquartile ranges of 29 years and 8.8 kg/m^2^, respectively. A total of 50.52% of the participants were female. Most of them were non-Hispanic White (63.84%), had an education above the college (62.24%), had high blood pressure (30.51%), or were non-gout (93.05%). Approximately half of the participants were married (53.26%) and were examined or sampled between May and October (55.49%). A total of 40.81% of them smoked more than 100 cigarettes during their life, and 37.54% of them consumed more than 12 alcohol drinks per year. Overall, 20.14% of people have a family income-to-poverty ratio higher than 1. A total of 42.15% of participants reported that their close relatives had diabetes. The median intake of energy was 2036.63 kcal/m^2^. The median protein, carbohydrate, and fat intake were 76.60, 234.99, and 79.70 gm, respectively. The medians of ALT and AST were 20.00 and 22.00 U/L, respectively. The median BUN and creatinine levels were 14.00 and 0.85 mg/dL, respectively. The median and quartiles of the CDAI were 0.00 and (−1.98, 2.55), respectively. All demographic characteristics of people with or without diabetes are presented in detail in Table 1. There were statistically significant differences in age, BMI, education, marital status, smoking status, blood pressure, gout status, family history of diabetes, energy intake, ALT, and BUN between people with and without diabetes.

**Table 1:** Demographic characteristics of participants by diabetes status among US adults, NHANES 2011–2018 (*n* = 2824).

Characteristic	Overall	No Diabetes	Diabetes	*P* value
Age (years)	48.00 (32.00, 61.00)	45.00 (31.00, 58.00)	62.00 (52.00, 69.00)	<0.001
Gender (%, SE)				0.295
Female	50.52 (1.45)	51.09 (1.60)	46.36 (4.01)	
Male	49.48 (1.45)	48.91 (1.60)	53.64 (4.01)	
BMI (kg/m^2^)	28.20 (24.30, 33.10)	27.80 (24.10, 32.40)	31.50 (27.90, 37.90)	<0.001
Race (%, SE)				0.052
Mexican American	8.79 (1.33)	8.71 (1.30)	9.38 (2.00)	
Non-Hispanic Black	11.00 (1.55)	10.46 (1.50)	14.92 (2.82)	
Non-Hispanic White	63.84 (2.46)	64.64 (2.43)	58.06 (4.19)	
Others	13.36 (1.25)	16.18 (1.30)	17.64 (1.98)	
Education (%, SE)				0.005
Below high school	11.19 (0.87)	10.32 (0.90)	17.52 (2.31)	
College and above	62.24 (1.76)	63.28 (1.94)	54.74 (4.15)	
High school	23.74 (1.28)	23.28 (1.37)	27.07 (3.35)	
Missing	2.83 (0.35)	3.13 (0.40)	0.68 (0.37)	
Marry status (%, SE)				<0.001
Divorced	9.82 (0.84)	9.40 (0.90)	12.91 (2.76)	
Living with partner	8.26 (0.89)	8.90 (1.02)	3.65 (0.89)	
Married	53.26 (1.87)	52.02 (2.03)	62.24 (3.86)	
Never married	18.14 (1.24)	19.47 (1.34)	8.52 (1.80)	
Refused	2.84 (0.35)	3.14 (0.39)	0.68 (0.37)	
Separated	2.67 (0.31)	2.71 (0.33)	2.40 (0.78)	
Widowed	5.00 (0.50)	4.37 (0.50)	9.60 (1.97)	
Ratio of family income to poverty (%, SE)				0.494
<1	79.86 (1.29)	80.20 (1.31)	77.80 (3.11)	
≥1	20.14 (1.29)	19.80 (1.31)	22.20 (3.11)	
Smoked ≥100 cigarettes in life (%, SE)				0.006
Yes	40.81 (1.23)	39.59 (1.34)	49.69 (3.14)	
No	59.19 (1.23)	60.41 (1.34)	50.31 (3.14)	
Had ≥12 alcohol drinks/1 yr (%, SE)				0.231
Yes	37.54 (2.07)	38.18 (2.32)	32.87 (3.05)	
No	11.32 (0.90)	11.00 (0.98)	13.66 (1.76)	
Missing	51.14 (1.98)	50.82 (2.21)	53.48 (3.01)	
Had high blood pressure (%, SE)				<0.001
Yes	30.51 (1.23)	24.98 (1.29)	70.59 (3.11)	
No	69.49 (1.23)	75.02 (1.29)	29.41 (3.11)	
Had gout (%, SE)				<0.001
Yes	4.06 (0.36)	2.57 (0.35)	14.88 (1.59)	<0.001
No	93.05 (0.46)	94.28 (0.48)	84.13 (1.74)	
Missing	2.90 (0.35)	3.16 (0.40)	0.98 (0.44)	
Close relative had diabetes (%, SE)				<0.001
Yes	42.15 (1.23)	38.65 (1.30)	67.53 (3.56)	
No	53.27 (1.40)	56.62 (1.51)	28.98 (3.25)	
Missing	4.58 (0.54)	4.73 (0.56)	3.49 (1.13)	
Time of examination/Sampling (%, SE)				0.515
May 1 through October 31	55.49 (5.32)	55.20 (5.26)	57.60 (6.62)	
November 1 through April 30	44.51 (5.32)	44.80 (5.26)	42.40 (6.62)	
Energy (kcal)	2036.63 (1531.81, 2655.00)	2055.00 (1559.00, 2683.84)	1921.49 (1436.18, 2541.91)	0.041
Protein (gm)	76.60 (54.58, 103.42)	77.15 (54.90, 104.09)	74.61 (52.54, 96.36)	0.198
Carbohydrate (gm)	234.99 (165.40, 312.30)	238.55 (166.12, 316.53)	214.90 (157.00, 290.44)	0.075
Total fat (gm)	79.70 (55.74, 110.66)	79.70 (55.70, 110.50)	79.42 (56.41, 111.23)	0.886
ALT (U/L)	20.00 (15.00, 28.00)	20.00 (15.00, 28.00)	21.00 (16.00, 31.00)	0.048
AST (U/L)	22.00 (18.00, 26.00)	22.00 (18.00, 26.00)	22.00 (17.27, 27.00)	0.893
Blood urea nitrogen (mg/dL)	14.00 (11.00, 17.00)	14.00 (11.00, 17.00)	16.00 (13.00, 20.00)	<0.001
Creatinine (mg/dL)	0.85 (0.71, 0.98)	0.84 (0.71, 0.97)	0.90 (0.75, 1.05)	<0.001
CDAI (continuous)	0.00 (−1.98, 2.55)	0.03 (−1.90, 2.57)	−0.35 (−2.30, 2.10)	0.286
Low	73.25 (1.51)	72.90 (1.56)	75.50 (3.40)	0.477
High	26.75 (1.51)	27.10 (1.56)	24.50 (3.40)	

*SE* standard error, *BMI* body mass index, *ALT* alanine aminotransferase, *AST* aspartate aminotransferase, *CDAI* comprehensive dietary antioxidant index.

### Concentrations of contaminated metabolites

All concentrations of contaminated metabolites are shown in Table 2. The exposure levels of OPEs are lower than those of PAEs. Among the groups of PAEs, the MEP had the highest value, with a median of 26.081 µg/g Cr, followed by MBP (median 8.380 µg/g Cr), MECPP (median 7.066 µg/g Cr), and MiBP (median 6.908 µg/g Cr). The MCPP had the lowest median concentration (0.968 µg/g Cr). Among the OPE metabolites, BDCP had the highest concentration, with a median of 1.068 µg/g Cr, followed by DPHP, with a median concentration of 0.747 µg/g Cr. DBUP was detected at the lowest concentration, with a median of 0.108 µg/g Cr. Except for the greater correlations between metabolites of DEHP, such as MEHHP, MEOHP, and MECPP, all the other correlations showed low to medium levels (Fig. S3).

**Table 2:** Urinary concentrations of plastic additive metabolites, NHANES 2011–2018 (μg/g creatinine).

Chemical	Percentiles				
	5th	25th	50th	75th	95th
MEP	5.462	12.600	26.081	63.968	349.191
MnBP	2.632	5.367	8.380	13.519	30.198
MiBP	2.263	4.394	6.908	11.194	26.476
MBzP	0.671	1.622	3.023	6.402	22.133
MEHHP	1.493	2.865	4.583	7.320	18.244
MEOHP	0.886	1.786	2.941	4.630	11.250
MECPP	2.375	4.658	7.066	11.111	24.912
MCPP	0.311	0.596	0.968	1.574	4.907
MCNP	0.414	0.821	1.338	2.288	6.364
MCOP	1.592	2.951	4.848	10.000	54.310
BCPP	0.035	0.075	0.151	0.320	0.982
BCEP	0.050	0.180	0.381	0.833	2.793
BDCP	0.203	0.554	1.068	1.883	5.375
DBUP	0.036	0.066	0.108	0.197	0.548
DPHP	0.228	0.455	0.747	1.356	5.082

*MEP* mono-ethyl phthalate, *MnBP* mono-*n*-butyl phthalate, *MiBP* mono-isobutyl phthalate, *MBzP* monobenzyl phthalate, *MEHHP* mono-(2-ethyl-5-hydroxyhexyl) phthalate, *MEOHP* mono-(2-ethyl-5-oxohexyl) phthalate, *MECPP* mono-2-ethyl-5-carboxypentyl phthalate, *MCPP* mono-(3-carboxypropyl) phthalate, *MCNP* mono (carboxynonyl) phthalate, *MCOP* mono (carboxyoctyl) phthalate, *BCPP* bis(1-chloro-2-propyl) phosphate, *BCEP* bis(2-chloroethyl) phosphate, *BDCP* bis-*p*-cresyl phosphate, *DBUP* dibutyl phosphate, *DPHP* diphenyl phosphate.

### Association between each contaminating metabolite and diabetes incidence status

In the first scenario, individuals with a borderline clinical diagnosis and clinical diagnosis of diabetes were combined into the diabetes group, and exposure to most PAE metabolites, such as MEP, MnBP, MEHHP, MEOHP, MECPP, and MCNP, was associated with an increased risk of diabetes according to the crude models. The BDCP concentration in OPEs is also positively correlated with the risk of diabetes. After we adjusted for demographic variables such as age and sex, these associations became statistically insignificant. However, after further adjusting for CDAI components, the association between PAE metabolite exposure and increased risk of diabetes was marginally statistically significant (Table S3).

In the second scenario, where individuals with borderline clinical diagnosis of diabetes were included in the normal population, the observed associations between exposure to additive metabolites and the risk of diabetes were similar (Table S4).

### Association between contaminated metabolite mixture and diabetes

In both the first and second scenarios, for every 1-SD increase in ERS, the risk of diabetes increased by 25 and 21%, respectively (Table 3). BCEP and BCPP provided the highest weights for the positive relationships. The other four substances that provided higher weights were DBUP, BDCP, MCNP, and MEOHP (Fig. S2b, c).

**Table 3:** Associations of mixture exposure to PAEs and OPEs with diabetes, NHANES 2011–2018.

Outcome	Exposure	OR (95%CI)	*P* value	*P* for trend
First scenario diabetes	ERS			
	Continuous	**1.25 (1.13, 1.39)**	**<0.001**	—
	T1	Reference	—	**<0.001**
	T2	1.17 (0.87, 1.58)	0.310	
	T3	**1.77 (1.32, 2.37)**	**<0.001**	
	ERS_CV			
	Continuous	1.08 (0.97, 1.19)	0.105	—
	T1	Reference	—	**0.005**
	T2	1.15 (0.86, 1.55)	0.353	
	T3	**1.49 (1.12, 1.99)**	**0.007**	
Second scenario diabetes	ERS			
	Continuous	**1.21 (1.09, 1.34)**	**<0.001**	—
	T1	Reference	—	**<0.001**
	T2	**1.41 (1.01, 1.97)**	**0.043**	
	T3	**1.97 (1.43, 2.73)**	**<0.001**	
	ERS_CV			
	Continuous	**1.09 (0.98, 1.20)**	**0.079**	—
	T1	Reference	—	**0.011**
	T2	1.14 (0.83, 1.57)	0.353	
	T3	**1.48 (1.08, 2.02)**	**0.014**	

*PAEs* phthalate esters, *OPEs* organic phosphate esters, *ERS* environmental risk score, *ERS_CV* environmental risk score by cross-validation approach.

Bold values indicate statistical significance.

Compared with the lowest tertile array of ERS, the middle tertile array of ERS increased the risk of diabetes by 17 and 41%, while the highest tertile array of ERS increased the risk of diabetes by 1.77 times and 1.97 times, respectively. A chi-square test for trend indicated that exposure to the additive mixture (ERS) was positively associated with an increased risk of diabetes in a linear manner. The sensitivity analysis results of the relationships between diabetes risk and the ERS constructed by the cross-validation method were also similar (Table 3).

### Modifying effects of the CDAI on the associations between ERS exposure and diabetes risk

The *p* values for the interaction showed that there was an interaction between the ERS and the CDAI. Regardless of the scenario or CDAI, ERS, and the CDAI had a positive interaction effect on the risk of diabetes, with values ranging from 9 to 90%. Interestingly, in the high CDAI subgroup, the association between ERS and diabetes risk was lower than that in the low CDAI subgroup (Table 4).

**Table 4:** Modifying effects of the CDAI on the associations of mixed exposure to PAEs and OPEs with diabetes, NHANES 2011–2018.

Outcome	Exposure	CDAI	OR (95%CI)	*P* value	*P* for interaction
First Scenario Diabetes	ERS	Low	**1.83 (1.37, 2.55)**	**<0.001**	**0.038**
		High	**1.28 (1.15, 1.45)**	**<0.001**	
	ERS_CV	Low	**1.42 (1.08, 1.98)**	**0.025**	0.105
		High	1.09 (0.99, 1.21)	0.094	
Second Scenario Diabetes	ERS	Low	**1.90 (1.41, 2.68)**	**<0.001**	**0.009**
		High	**1.21 (1.09, 1.36)**	**<0.001**	
	ERS_CV	Low	**1.49 (1.11, 2.13)**	**0.017**	0.069
		High	1.08 (0.98, 1.21)	0.108	

*PAEs* phthalate esters, *OPEs* organic phosphate esters, *ERS* environmental risk score, *ERS_CV* environmental risk score by cross-validation approach, *CDAI* composite dietary antioxidant index.

Bold values indicate statistical significance.

According to the quantile g-computation models, without adjusting for confounding factors, the risk of diabetes increased by 27.4 and 36.0% when all metabolites increased by one quantile simultaneously (Table 5). When adjusting for confounding factors, the risk of diabetes increased by 1.285 times and 1.393 times when all metabolites increased by one quantile simultaneously. According to both the coarse model and the adjusted model, when both CDAI components and additive metabolites are included in the quantile g-computation, the risk of diabetes is no longer significantly increased when all metabolites (protecting CDAI components) increase by one quantile simultaneously. Overall, MECPP and MCNP provided the greatest positive weights, while vitamin C and zinc provided the greatest negative weights (Fig. S4, S5).

**Table 5:** The association between coexposure to PAEs and OPEs and diabetes from quatile g-computation, NHANES 2011–2018.

Outcome	Models	Included CDAI components	psi1 (95% CIs)	*t*	*P* value
First scenario diabetes	Crude	No	**1.274 (1.053, 2.867)**	**2.493**	**0.013**
	Crude	Yes	1.086 (0.849, 2.337)	0.654	0.513
	Adjusted	No	**1.285 (1.016, 2.763)**	**2.095**	**0.036**
	Adjusted	Yes	1.324 (0.916, 2.500)	1.494	0.135
Second scenario diabetes	Crude	No	**1.360 (1.107, 3.026)**	**2.933**	**0.003**
	Crude	Yes	1.130 (0.867, 2.379)	0.903	0.367
	Adjusted	No	**1.393 (1.083, 2.954)**	**2.583**	**0.010**
	Adjusted	Yes	1.334 (0.898, 2.455)	0.153	1.428

*PAEs* phthalate esters, *OPEs* organic phosphate esters, *CDAI* component dietary antioxidant index.

Bold values indicate statistical significance.

### Potential mechanisms

For TCPP, 17 genes and three phenotypes were identified in relation to T2DM (Fig. 1A, B). After merging these genes with TCPP-gene-T2DM, four genes associated with T2DM were identified: CPT1A (degree = 3), PPARA (degree = 2), PPARG (degree = 1), and FGF21 (degree = 1). The top two strongest genes (CPT1A and PPARA) and their related three phenotypes (GO: 0009410; GO: 0032000; GO: 0006006) were considered potential molecular initiating events (MIEs) and key events (KEs) in the TCPP-induced AOP for T2DM, respectively. Therefore, a putative AOP for TCPP-associated effects on T2DM was proposed, the sequence of which is described below. When exposed to TCPP, the CPT1A gene or PPARA gene is activated, resulting in an enhanced response to xenobiotic stimulus (GO: 0009410), which positively regulates the fatty acid beta-oxidation (GO: 0032000), and further changes in glucose metabolic process (GO: 0006006), which eventually leads to T2DM (Fig. 1D).

For TCEP, a total of 36 genes and 11 phenotypes were identified in relation to T2DM (Fig. 1A, C). After merging these genes with TCEP-gene-T2DM, 14 target genes were found to be associated with T2DM (Fig. 1D). Among these genes, the two genes with the strongest association with T2DM were AKT1 (degree = 6) and HNF4A (degree = 5) (Fig. 1E). These two genes and their ten related phenotypes (hsa04910; GO: 0032869; GO: 0042593; GO:0050796; GO:0009749; GO:0030073; GO:0009410; GO:0006006; GO: 0031016) are considered as MIEs and KEs, respectively, in the TCEP-induced AOP for T2DM. Therefore, a putative AOP network (Fig. 1D) for TCEP-associated effects on T2DM was proposed with a sequence of events described below. When exposed to TCEP, the AKT1A or HNF4A genes are activated in response to exogenous stimuli (GO: 0009410) or in response to glucose (GO: 0009749) and then positively regulate the process of glucose metabolism (GO: 0010907), increase the cellular response to insulin stimulus (GO: 0032869), or regulate insulin secretion (GO: 0050796), further disturbing the process of glucose metabolism (GO: 0006006) or insulin secretion (GO: 0030073) to destroy glucose homeostasis (GO: 0042593) or pancreas development (GO: 0031016); these changes ultimately lead to the development of T2DM. Another possibility is that the activation of the AKT1A or HNF4A gene leads to the development of T2DM through the aberrant insulin signaling pathway (hsa04910).

## Discussion

In summary, the risk of diabetes is significantly increased when individuals are exposed to a mixture of PAE and OPE metabolites, with BCEP, BCPP, MCNP, and MECPP being key active substances. The simultaneous consumption of antioxidant foods, especially foods containing vitamin C and zinc, can alleviate the risk of diabetes caused by PAEs and OPEs. Previous studies have not investigated the association between mixed exposure to PAEs or OPEs and the risk of diabetes, but harmful effects on other health issues, such as adverse reproductive outcomes [11], childhood asthma [10], and neurodevelopmental deficits [12], have been reported.

Regarding exposure to PAEs alone, data from the Korean National Environmental Health Survey (KoNEHS) showed significant positive associations between diabetes and PAEs, and ORs of the highest quartile were for DEHP (2.49, 95% CI = 1.43–4.33), MEHHP (3.03, 95% CI = 1.69–5.45), MEOHP (1.74, 95% CI = 1.03–2.92), MECCP (1.67, 95% CI = 1.02–2.73), MnBP (2.17, 95% CI = 1.28–3.66), MBzP (1.89, 95% CI = 1.11–3.22), and MCPP (2.01, 95% CI = 1.19–3.42) compared to the first quartile of PAEs [13]. A case‒control study in China revealed significant positive associations between urinary concentrations of the most studied PAE metabolites and T2DM, with odd ratios indicating extreme PAE quartiles ranging from 2.09 to 40.53, whereas two secondary metabolites, MECPP and mono[(2-carboxymethyl)hexyl] phthalate (MCMHP), showed significant inverse relationships with T2DM [34]. Women with higher levels of mono-*n*-butyl phthalate (MnBP), mono-isobutyl phthalate (MiBP), monobenzyl phthalate (MBzP), mono-(3-carboxypropyl) phthalate (MCPP), and three di-(2-ethylhexyl) phthalate metabolites (ΣDEHP) had greater odds of diabetes than women with the lowest levels of these phthalates. Women in the highest quartile for MBzP and MiBP had almost twice the odds of having diabetes [OR = 1.96 (95% CI: 1.11, 3.47) and OR = 1.95 (95% CI: 0.99, 3.85), respectively] compared with women in the lowest quartile [35]. At present, there is insufficient evidence about the association between combined exposure to multiple PAE metabolites and diabetes [36].

For OPE exposure alone, according to the data from the NHANES 1999-2008 and 2011-2012, DMTP was significantly associated with higher levels of serum insulin (β = 0.21, 95% CI = 0.06-0.36) and HOMA-IR (β = 0.08, 95% CI = 0.02-0.14) and increased odds of T2DM (OR = 1.05, 95% CI = 1.01–1.08). Other OPEs were not significantly associated with serum markers of glucose homeostasis or T2DM [15]. Evidence from the China BAPE Study [14] demonstrated that exposure to five OPE mixtures (trimethylolpropane phosphate, triphenyl phosphate, tri-iso-butyl phosphate, dibutyl phosphate, and diphenyl phosphate) was associated with increased levels of glycometabolic markers [14]. A comparison between these research results and the results of this study is difficult. The demographic characteristics and OPE exposure levels differed, as did the sample size, study design and statistical analysis methods. For instance, all the evidence, including this study, comes from cross-sectional surveys or case‒control studies, which are designed to make it difficult to infer causality. More prospective research evidence is needed in the future to explore the causal relationships between PAE and OPE exposure and diabetes.

However, the biological mechanism underlying the increased risk of diabetes caused by these plastic additives is still unclear. By mining public database resources and bioinformatics analysis, we proposed that two key substances, TCPP and TCEP, may lead to AOP in T2DM, suggesting that the enhanced response to exogenous substances regulates fatty acid beta-oxidation or glucose metabolism, further disrupting glucose metabolism disorders and ultimately leading to T2DM. In addition, abnormalities in the insulin signaling pathway may also be one of the possible mechanisms. This finding is consistent with previous findings [14]. This putative AOP model was proposed based on the data from multiomics analyses. It has been suggested that triggers of molecular initiation events (e.g., insulin receptor and glucose transporter type 4) with subsequent key events, including disruptions in signal transduction pathways (e.g., phosphatidylinositol 3-kinase/protein kinase B and insulin secretion signaling) and biological functions (glucose uptake and insulin secretion), may constitute the diabetogenic effects of OPEs [14].

Another possible mechanism is oxidative stress and inflammation. Increased oxidative stress and inflammation can lead to insulin resistance, impaired insulin secretion, and, ultimately, T2DM [37–39]. The inflammatory response and metabolic regulation are highly coordinated because abnormal inflammatory function can lead to a series of chronic metabolic diseases, including T2DM [40]. Phthalates are thought to be important environmental risk factors that may influence inflammation and oxidative stress [41–43]. Multiple metabolites of organophosphate flame retardants and their mixed exposures have also been found to be associated with elevated levels of biomarkers of oxidative stress [44]. An animal experiment showed that TCPP induced the overexpression of adipogenic genes and inhibited the expression of fatty acid β-oxidation genes. Excessive lipid synthesis and insufficient consumption can damage the antioxidant system and lead to the overexpression of fatty acid β-oxidation proinflammatory cytokines, which triggers oxidative damage and inflammation [45]. These findings seem to coincide with the results of this study. This study identified fatty acid beta-oxidation as one of the important phenotypes associated with potential TCPP-associated AOP in patients with T2DM. In addition, in the TCEP-induced AOP, the target genes also include IL6, PPARA, and PPARGC1A, which are closely related to inflammation and oxidative stress. These changes may also constitute the mechanism by which the antioxidant foods found in this study mitigate the increased risk of diabetes from mixed exposure to plastic additives.

There are still many shortcomings that need to be taken into consideration in this study. First, this was a cross-sectional survey study design, which has the inherent drawback of not being able to determine the chronological order of exposure and outcome and cannot account for the causal relationship between exposure to plastic additives and diabetes. Longitudinal studies would be more suitable to establish temporal relationships between exposure to plastic additives and the development of diabetes. Second, there may still be unmeasured confounding variables that could influence the observed associations. Factors such as dietary habits, lifestyle factors, genetic predispositions were not fully accounted. Third, in addition to plasticizers and flame retardants, plastic products contain other additives, polymer matrices, degradation products, and adsorbed pollutants that pose a threat to human health. Future research should aim to explore a wider range of plastic product chemicals to better understand their health effects. Forth, the type of diabetes in this study was not detailed as to whether it was type 1 or type 2. The pathogenesis of these two types of diabetes may be different [46, 47], so this limits the study’s ability to draw specific conclusions about the association between plastic additives and each type of diabetes. Finally, we utilized two commonly used adaptive elastic network models and quantile g computation models to evaluate the effects of mixed chemical exposure. Although the results of these two studies are consistent and suggest that mixed exposure to PAEs and OPEs is associated with an increased risk of diabetes, slight differences in the key substances were found. This approach is not conducive to formulating further precise intervention measures or informing the direction of policy reform.

## Conclusion

In conclusion, the combination of plastic plasticizers and flame retardants, such as PAEs and OPEs, increases the risk of developing diabetes. Special attention should be given to reducing contact and exposure to parent chemicals such as TCEP (the parent chemical of BCEP), TCPP (the parent chemical of BCPP), DCNP (the parent chemical of MCNP), and DEHP (the parent chemical of MECPP). It is recommended that individuals consume more antioxidant foods, such as vitamin C and zinc, which may reduce the risk of diabetes caused by exposure to plastic additives. In the future, it is necessary to incorporate a wider range of plastic product chemicals to explore the impact of plastic pollution on human health.

## Supplementary information

Table Of Supplementary Contents

figure s1

figure s2

figure s3

figure s4

figure s5

## Data Availability

The datasets generated during and/or analysed during the current study are available in the NHANES website (https://www.cdc.gov/nchs/nhanes/index.htm).
