# Supplementary material for: Potential mechanisms and modifications of dietary antioxidants on the associations between co-exposure to plastic additives and diabetes
Source: Nutr Diabetes. 2024 Sep 3;14:72. doi: 10.1038/s41387-024-00330-1 (PMC11372220; doi:10.1038/s41387-024-00330-1)
Supplement: Supplementary file 1 — Table Of Supplementary Contents [file 41387_2024_330_MOESM1_ESM.docx]

**TABLE OF CONTENTS**

[Table S1. Missingness of metabolites of phthalates and organophosphate esters, NHANES 2011-2018 2](#_Toc22677)

[Table S2. Summary of the collected genes and phenotypes 4](#_Toc27936)

[Table S3. Associations between metabolites and diabetes in single-pollutant models in the first scenario, NHANES 2011-2018 5](#_Toc7286)

[Table S4. Associations between metabolites and diabetes in single-pollutant models in the second scenario, NHANES 2011-2018 7](#_Toc19456)

[Supplementary Figure Legends 9](#_Toc14187)

[Figure S1. Flow diagram of participant selection. 9](#_Toc12031)

[Figure S2. The construction of ERS via adaptive elastic net model. 9](#_Toc25336)

[Figure S3. Spearman correlations between plastic additive metabolites. 9](#_Toc24164)

[Figure S4. Weights representing the proportion of positive or negative partial effects for each metabolite in the quantile g-computation models in the first scenario of diabetes. 9](#_Toc12234)

[Figure S5. Weights representing the proportion of positive or negative partial effects for each metabolite in the quantile g-computation models in the second scenario of diabetes. 10](#_Toc4996)

# Table S1. Missingness of metabolites of phthalates and organophosphate esters, NHANES 2011-2018

| **Metabolite** | **Missing rate** |
| --- | --- |
| MEP | 0.41% |
| MnBP | 0.68% |
| MiBP | 1.88% |
| MBzP | 2.86% |
| MEHP | 39.29% |
| MEHHP | 0.70% |
| MEOHP | 0.68% |
| MECPP | 0.18% |
| MCPP | 17.06% |
| MCNP | 2.88% |
| MCOP | 0.50% |
| MHNCH | 32.61% |
| MiNP | 76.77% |
| BCPP | 27.92% |
| BCEP | 10.91% |
| BDCP | 1.37% |
| DBUP | 24.32% |
| DPHP | 1.55% |
| TBBA | 40.77% |

**Abbreviations:** MEP, [mono-ethyl phthalate;](https://wwwn.cdc.gov/Nchs/Nhanes/2013-2014/PHTHTE_H.htm#URXMEP) MnBP, [mono-n-butyl phthalate](https://wwwn.cdc.gov/Nchs/Nhanes/2013-2014/PHTHTE_H.htm#URXMBP); MiBP, [mono-isobutyl phthalate;](https://wwwn.cdc.gov/Nchs/Nhanes/2013-2014/PHTHTE_H.htm#URXMIB)MBzP, [mono-benzyl phthalate](https://wwwn.cdc.gov/Nchs/Nhanes/2013-2014/PHTHTE_H.htm#URXMZP); MEHP, mono-(2-ethyl-hexyl) phthalate; MEHHP, mono-(2-ethyl-5-hydroxyhexyl) phthalate; MEOHP, Mono-(2-ethyl-5-oxohexyl) phthalate; MECPP, mono-2-ethyl-5-carboxypentyl phthalate; MCPP, [mono-(3-carboxypropyl) phthalate;](https://wwwn.cdc.gov/Nchs/Nhanes/2013-2014/PHTHTE_H.htm#URXMC1) MCNP, [mono (carboxynonyl) phthalate](https://wwwn.cdc.gov/Nchs/Nhanes/2013-2014/PHTHTE_H.htm#URXCNP); MCOP, [mono (carboxyoctyl) phthalate](https://wwwn.cdc.gov/Nchs/Nhanes/2013-2014/PHTHTE_H.htm#URXCOP); MNHCH, cyclohexane 1,2-dicarboxylic acid monohydroxy isononyl ester; MiNP, monoisononyl phthalate; BCPP, bis(1-chloro-2-propyl) phosphate; BCEP, bis(2-chloroethyl) phosphate; BDCP, bis-p-cresyl phosphate; DBUP, dibutyl phosphate; DPHP, diphenyl phosphate; TBBA, 2,3,4,5-tetrabromobenzoic acid.

# Table S2. Summary of the collected genes and phenotypes

| **Chemical/Disease** | **Subtype** | **CTD** | **DisGeNET** | **MalaCards** |
| --- | --- | --- | --- | --- |
| TCEP | Gene | 136 | — | — |
|  | Phenotype | 956: GO term  120: Pathway | — | — |
|  | Gene associated with T2DM | 16 | — | — |
| TCPP | Gene | 60 | — | — |
|  | Phenotype | 431: GO term  35: Pathway | — | — |
|  | Gene associated with T2DM | 5 | — | — |
| T2DM | Gene | — | 3134 | 594 |
|  | Phenotype | — | — | 35: GO term  8: Pathway |

**Abbreviations:** PAEs, phthalate esters; OPEs, Organic phosphate esters; TCEP, tris(2-chloroethyl) phosphate; TCPP, tris(1-chloro-2-propyl) phosphate; T2DM, type 2 diabetes mellitus; CTD, Comparative Toxicogenomics Database; DisGeNET, Disease Gene Network; GO, Gene Ontology.

# Table S3. Associations between metabolites and diabetes in single-pollutant models in the first scenario, NHANES 2011-2018

| **Metabolite** | **Crude model** | |  | **Model 1** |  |  | **Model 2** | |
| --- | --- | --- | --- | --- | --- | --- | --- | --- |
|  | **OR (95% CIs)** | **P value** |  | **OR (95% CIs)** | **P value** |  | **OR (95% CIs)** | **P value** |
| MEP | **1.166 (1.062, 1.28)** | **0.002** |  | 1.124 (0.942, 1.342) | 0.126 |  | **1.158 (1.006, 1.333)** | **0.044** |
| MnBP | **1.435 (1.224, 1.683)** | **<0.001** |  | **1.283 (0.915, 1.798)** | **0.100** |  | **1.278 (0.972, 1.681)** | **0.070** |
| MiBP | 1.134 (0.95, 1.354) | 0.158 |  | 1.228 (0.873, 1.727) | 0.151 |  | 1.242 (0.939, 1.645) | 0.103 |
| MBzP | 1.02 (0.887, 1.173) | 0.772 |  | 0.950 (0.719, 1.256) | 0.600 |  | 1.017 (0.812, 1.272) | 0.858 |
| MEHHP | **1.369 (1.142, 1.64)** | **0.001** |  | 1.219 (0.884, 1.681) | 0.145 |  | **1.285 (0.972, 1.698)** | **0.069** |
| MEOHP | **1.403 (1.18, 1.669)** | **<0.001** |  | 1.266 (0.919, 1.744) | 0.101 |  | **1.312 (0.993, 1.733)** | **0.054** |
| MECPP | **1.492 (1.256, 1.773)** | **<0.001** |  | **1.297 (0.919, 1.829)** | **0.095** |  | **1.340 (0.993, 1.809)** | **0.054** |
| MCPP | 1.114 (0.946, 1.313) | 0.187 |  | 0.956 (0.749, 1.221) | 0.601 |  | 0.97 (0.783, 1.202) | 0.728 |
| MCNP | **1.146 (0.984, 1.334)** | **0.077** |  | 1.146 (0.895, 1.467) | 0.177 |  | 1.122 (0.912, 1.38) | 0.213 |
| MCOP | 1.015 (0.917, 1.124) | 0.765 |  | 0.966 (0.778, 1.2) | 0.649 |  | 0.968 (0.807, 1.161) | 0.666 |
| BCPP | 1.077 (0.931, 1.247) | 0.306 |  | 1.011 (0.749, 1.365) | 0.916 |  | 1.031 (0.81, 1.313) | 0.759 |
| BCEP | 1.044 (0.87, 1.251) | 0.635 |  | 0.998 (0.725, 1.373) | 0.982 |  | 1.059 (0.839, 1.337) | 0.555 |
| BDCP | **0.873 (0.764, 0.997)** | **0.045** |  | 1.021 (0.783, 1.331) | 0.822 |  | 1.031 (0.84, 1.264) | 0.720 |
| DBUP | 1.024 (0.923, 1.136) | 0.644 |  | 0.978 (0.802, 1.194) | 0.751 |  | 1.007 (0.847, 1.197) | 0.922 |
| DPHP | 0.96 (0.832, 1.107) | 0.560 |  | 1.015 (0.754, 1.367) | 0.882 |  | 0.969 (0.756, 1.241) | 0.754 |

**Note:** Model 1 adjusted for age, gender, BMI, race, education, marry status, ratio of family income to poverty, smoking, drinking, blood pressure, gout, family history of diabetes, and time of examination/sampling; Model 2 additionally adjusted for energy, protein, carbohydrate, total fat, ALT, AST, blood urea nitrogen and creatinine.

**Abbreviations:** MEP, [mono-ethyl phthalate;](https://wwwn.cdc.gov/Nchs/Nhanes/2013-2014/PHTHTE_H.htm#URXMEP) MnBP, [mono-n-butyl phthalate](https://wwwn.cdc.gov/Nchs/Nhanes/2013-2014/PHTHTE_H.htm#URXMBP); MiBP, [mono-isobutyl phthalate;](https://wwwn.cdc.gov/Nchs/Nhanes/2013-2014/PHTHTE_H.htm#URXMIB) MBzP, [mono-benzyl phthalate](https://wwwn.cdc.gov/Nchs/Nhanes/2013-2014/PHTHTE_H.htm#URXMZP); MEHHP, mono-(2-ethyl-5-hydroxyhexyl) phthalate; MEOHP, Mono-(2-ethyl-5-oxohexyl) phthalate; MECPP, mono-2-ethyl-5-carboxypentyl phthalate; MCPP, [mono-(3-carboxypropyl) phthalate;](https://wwwn.cdc.gov/Nchs/Nhanes/2013-2014/PHTHTE_H.htm#URXMC1) MCNP, [mono (carboxynonyl) phthalate](https://wwwn.cdc.gov/Nchs/Nhanes/2013-2014/PHTHTE_H.htm#URXCNP); MCOP, [mono (carboxyoctyl) phthalate](https://wwwn.cdc.gov/Nchs/Nhanes/2013-2014/PHTHTE_H.htm#URXCOP); BCPP, bis(1-chloro-2-propyl) phosphate; BCEP, bis(2-chloroethyl) phosphate; BDCP, bis-p-cresyl phosphate; DBUP, dibutyl phosphate; and DPHP, diphenyl phosphate; OR, odds ratio; CI, confidence interval.

# Table S4. Associations between metabolites and diabetes in single-pollutant models in the second scenario, NHANES 2011-2018

| **Metabolite** | **Crude model** | |  | **Model 1** |  |  | **Model 2** | |
| --- | --- | --- | --- | --- | --- | --- | --- | --- |
|  | **OR (95% CIs)** | **P value** |  | **OR (95% CIs)** | **P value** |  | **OR (95% CIs)** | **P value** |
| MEP | **1.17 (1.048, 1.306)** | **0.007** |  | 1.127 (0.917, 1.385) | 0.162 |  | **1.155 (0.967, 1.379)** | **0.091** |
| MnBP | **1.581 (1.345, 1.858)** | **<0.001** |  | **1.442 (1.049, 1.983)** | **0.035** |  | **1.426 (1.09, 1.866)** | **0.019** |
| MiBP | 1.144 (0.934, 1.401) | 0.186 |  | 1.228 (0.819, 1.842) | 0.205 |  | 1.246 (0.896, 1.734) | 0.147 |
| MBzP | 0.998 (0.852, 1.169) | 0.975 |  | 0.91 (0.658, 1.259) | 0.423 |  | 0.977 (0.759, 1.258) | 0.823 |
| MEHHP | **1.416 (1.187, 1.688)** | **<0.001** |  | 1.235 (0.903, 1.689) | 0.121 |  | **1.325 (0.998, 1.759)** | **0.051** |
| MEOHP | **1.464 (1.234, 1.735)** | **<0.001** |  | **1.299 (0.956, 1.764)** | **0.073** |  | **1.363 (1.031, 1.802)** | **0.036** |
| MECPP | **1.586 (1.315, 1.912)** | **<0.001** |  | **1.372 (0.948, 1.984)** | **0.072** |  | **1.439 (1.033, 2.003)** | **0.037** |
| MCPP | **1.172 (0.998, 1.377)** | **0.053** |  | 1.001 (0.788, 1.273) | 0.986 |  | 1.013 (0.815, 1.258) | 0.888 |
| MCNP | **1.241 (1.054, 1.461)** | **0.011** |  | **1.307 (0.999, 1.711)** | **0.051** |  | **1.254 (0.978, 1.609)** | **0.066** |
| MCOP | 1.042 (0.911, 1.193) | 0.536 |  | 0.993 (0.751, 1.312) | 0.941 |  | 0.994 (0.787, 1.257) | 0.953 |
| BCPP | 1.067 (0.914, 1.246) | 0.400 |  | 0.979 (0.714, 1.343) | 0.844 |  | 1.005 (0.781, 1.292) | 0.965 |
| BCEP | 1.017 (0.868, 1.193) | 0.825 |  | 0.947 (0.694, 1.294) | 0.620 |  | 1.015 (0.796, 1.296) | 0.878 |
| BDCP | 0.889 (0.76, 1.04) | 0.136 |  | 1.085 (0.797, 1.475) | 0.463 |  | 1.092 (0.867, 1.377) | 0.372 |
| DBUP | 1.071 (0.941, 1.219) | 0.289 |  | 1.013 (0.775, 1.325) | 0.886 |  | 1.052 (0.86, 1.286) | 0.548 |
| DPHP | 1.032 (0.905, 1.176) | 0.627 |  | 1.129 (0.875, 1.457) | 0.226 |  | 1.062 (0.859, 1.312) | 0.502 |

**Note:** Model 1 adjusted for age, gender, BMI, race, education, marry status, ratio of family income to poverty, smoking, drinking, blood pressure, gout, family history of diabetes, and time of examination/sampling; Model 2 additionally adjusted for energy, protein, carbohydrate, total fat, ALT, AST, blood urea nitrogen and creatinine.

**Abbreviations:** MEP, [mono-ethyl phthalate;](https://wwwn.cdc.gov/Nchs/Nhanes/2013-2014/PHTHTE_H.htm#URXMEP) MnBP, [mono-n-butyl phthalate](https://wwwn.cdc.gov/Nchs/Nhanes/2013-2014/PHTHTE_H.htm#URXMBP); MiBP, [mono-isobutyl phthalate;](https://wwwn.cdc.gov/Nchs/Nhanes/2013-2014/PHTHTE_H.htm#URXMIB) MBzP, [mono-benzyl phthalate](https://wwwn.cdc.gov/Nchs/Nhanes/2013-2014/PHTHTE_H.htm#URXMZP); MEHHP, mono-(2-ethyl-5-hydroxyhexyl) phthalate; MEOHP, Mono-(2-ethyl-5-oxohexyl) phthalate; MECPP, mono-2-ethyl-5-carboxypentyl phthalate; MCPP, [mono-(3-carboxypropyl) phthalate;](https://wwwn.cdc.gov/Nchs/Nhanes/2013-2014/PHTHTE_H.htm#URXMC1) MCNP, [mono (carboxynonyl) phthalate](https://wwwn.cdc.gov/Nchs/Nhanes/2013-2014/PHTHTE_H.htm#URXCNP); MCOP, [mono (carboxyoctyl) phthalate](https://wwwn.cdc.gov/Nchs/Nhanes/2013-2014/PHTHTE_H.htm#URXCOP); BCPP, bis(1-chloro-2-propyl) phosphate; BCEP, bis(2-chloroethyl) phosphate; BDCP, bis-p-cresyl phosphate; DBUP, dibutyl phosphate; and DPHP, diphenyl phosphate; OR, odds ratio; CI, confidence interval.

# Supplementary Figure Legends

## Figure S1. Flow diagram of participant selection.

Figure S2. The construction of ERS via adaptive elastic net model. **A)** Five-fold cross-validation and optimization of the hyperparameters (lambda1 and lambda2) in the adaptive elastic net model. The red dot represents the optimal configuration of the hyperparameters. **B)** The coefficients of the adaptive elastic net model for each metabolite in the first scenario. **C)** The coefficients of the adaptive elastic net model for each metabolite in the second scenario. **Abbreviations:** ERS, environmental risk score; MEP, [mono-ethyl phthalate;](https://wwwn.cdc.gov/Nchs/Nhanes/2013-2014/PHTHTE_H.htm#URXMEP) MnBP, [mono-n-butyl phthalate](https://wwwn.cdc.gov/Nchs/Nhanes/2013-2014/PHTHTE_H.htm#URXMBP); MiBP, [mono-isobutyl phthalate;](https://wwwn.cdc.gov/Nchs/Nhanes/2013-2014/PHTHTE_H.htm#URXMIB) MBzP, [mono-benzyl phthalate](https://wwwn.cdc.gov/Nchs/Nhanes/2013-2014/PHTHTE_H.htm#URXMZP); MEHHP, mono-(2-ethyl-5-hydroxyhexyl) phthalate; MEOHP, Mono-(2-ethyl-5-oxohexyl) phthalate; MECPP, mono-2-ethyl-5-carboxypentyl phthalate; MCPP, [mono-(3-carboxypropyl) phthalate;](https://wwwn.cdc.gov/Nchs/Nhanes/2013-2014/PHTHTE_H.htm#URXMC1) MCNP, [mono (carboxynonyl) phthalate](https://wwwn.cdc.gov/Nchs/Nhanes/2013-2014/PHTHTE_H.htm#URXCNP); MCOP, [mono (carboxyoctyl) phthalate](https://wwwn.cdc.gov/Nchs/Nhanes/2013-2014/PHTHTE_H.htm#URXCOP); BCPP, bis(1-chloro-2-propyl) phosphate; BCEP, bis(2-chloroethyl) phosphate; BDCP, bis-p-cresyl phosphate; DBUP, dibutyl phosphate; and DPHP, diphenyl phosphate.

Figure S3. Spearman correlations between plastic additive metabolites. **Abbreviations:** MEP, [mono-ethyl phthalate;](https://wwwn.cdc.gov/Nchs/Nhanes/2013-2014/PHTHTE_H.htm#URXMEP) MnBP, [mono-n-butyl phthalate](https://wwwn.cdc.gov/Nchs/Nhanes/2013-2014/PHTHTE_H.htm#URXMBP); MiBP, [mono-isobutyl phthalate;](https://wwwn.cdc.gov/Nchs/Nhanes/2013-2014/PHTHTE_H.htm#URXMIB) MBzP, [mono-benzyl phthalate](https://wwwn.cdc.gov/Nchs/Nhanes/2013-2014/PHTHTE_H.htm#URXMZP); MEHHP, mono-(2-ethyl-5-hydroxyhexyl) phthalate; MEOHP, Mono-(2-ethyl-5-oxohexyl) phthalate; MECPP, mono-2-ethyl-5-carboxypentyl phthalate; MCPP, [mono-(3-carboxypropyl) phthalate;](https://wwwn.cdc.gov/Nchs/Nhanes/2013-2014/PHTHTE_H.htm#URXMC1) MCNP, [mono (carboxynonyl) phthalate](https://wwwn.cdc.gov/Nchs/Nhanes/2013-2014/PHTHTE_H.htm#URXCNP); MCOP, [mono (carboxyoctyl) phthalate](https://wwwn.cdc.gov/Nchs/Nhanes/2013-2014/PHTHTE_H.htm#URXCOP); BCPP, bis(1-chloro-2-propyl) phosphate; BCEP, bis(2-chloroethyl) phosphate; BDCP, bis-p-cresyl phosphate; DBUP, dibutyl phosphate; and DPHP, diphenyl phosphate.

Figure S4. Weights representing the proportion of positive or negative partial effects for each metabolite in the quantile g-computation models in the first scenario of diabetes. **A)** with covariate adjustment and no CDAI components; **B)** with covariate adjustment and CDAI components; **C)** with covariate adjustment in the CDAI low subgroup; **D)** with covariate adjustment in the CDAI high subgroup. **Abbreviations:** CDAI, component dietary antioxidant index; Vit, vitamin; MEP, [mono-ethyl phthalate;](https://wwwn.cdc.gov/Nchs/Nhanes/2013-2014/PHTHTE_H.htm#URXMEP) MnBP, [mono-n-butyl phthalate](https://wwwn.cdc.gov/Nchs/Nhanes/2013-2014/PHTHTE_H.htm#URXMBP); MiBP, [mono-isobutyl phthalate;](https://wwwn.cdc.gov/Nchs/Nhanes/2013-2014/PHTHTE_H.htm#URXMIB) MBzP, [mono-benzyl phthalate](https://wwwn.cdc.gov/Nchs/Nhanes/2013-2014/PHTHTE_H.htm#URXMZP); MEHHP, mono-(2-ethyl-5-hydroxyhexyl) phthalate; MEOHP, mono-(2-ethyl-5-oxohexyl) phthalate; MECPP, mono-2-ethyl-5-carboxypentyl phthalate; MCPP, [mono-(3-carboxypropyl) phthalate;](https://wwwn.cdc.gov/Nchs/Nhanes/2013-2014/PHTHTE_H.htm#URXMC1) MCNP, [mono (carboxynonyl) phthalate](https://wwwn.cdc.gov/Nchs/Nhanes/2013-2014/PHTHTE_H.htm#URXCNP); MCOP, [mono (carboxyoctyl) phthalate](https://wwwn.cdc.gov/Nchs/Nhanes/2013-2014/PHTHTE_H.htm#URXCOP); BCPP, bis(1-chloro-2-propyl) phosphate; BCEP, bis(2-chloroethyl) phosphate; BDCP, bis-p-cresyl phosphate; DBUP, dibutyl phosphate; and DPHP, diphenyl phosphate.

Figure S5. Weights representing the proportion of positive or negative partial effects for each metabolite in the quantile g-computation models in the second scenario of diabetes. **A)** with covariate adjustment and no CDAI components; **B)** with covariate adjustment and CDAI components; **C)** with covariate adjustment in the CDAI low subgroup; **D)** with covariate adjustment in the CDAI high subgroup. **Abbreviations:** CDAI, component dietary antioxidant index; Vit, vitamin; MEP, [mono-ethyl phthalate;](https://wwwn.cdc.gov/Nchs/Nhanes/2013-2014/PHTHTE_H.htm#URXMEP) MnBP, [mono-n-butyl phthalate](https://wwwn.cdc.gov/Nchs/Nhanes/2013-2014/PHTHTE_H.htm#URXMBP); MiBP, [mono-isobutyl phthalate;](https://wwwn.cdc.gov/Nchs/Nhanes/2013-2014/PHTHTE_H.htm#URXMIB) MBzP, [mono-benzyl phthalate](https://wwwn.cdc.gov/Nchs/Nhanes/2013-2014/PHTHTE_H.htm#URXMZP); MEHHP, mono-(2-ethyl-5-hydroxyhexyl) phthalate; MEOHP, mono-(2-ethyl-5-oxohexyl) phthalate; MECPP, mono-2-ethyl-5-carboxypentyl phthalate; MCPP, [mono-(3-carboxypropyl) phthalate;](https://wwwn.cdc.gov/Nchs/Nhanes/2013-2014/PHTHTE_H.htm#URXMC1) MCNP, [mono (carboxynonyl) phthalate](https://wwwn.cdc.gov/Nchs/Nhanes/2013-2014/PHTHTE_H.htm#URXCNP); MCOP, [mono (carboxyoctyl) phthalate](https://wwwn.cdc.gov/Nchs/Nhanes/2013-2014/PHTHTE_H.htm#URXCOP); BCPP, bis(1-chloro-2-propyl) phosphate; BCEP, bis(2-chloroethyl) phosphate; BDCP, bis-p-cresyl phosphate; DBUP, dibutyl phosphate; and DPHP, diphenyl phosphate.
