# Supplementary figures and images for: Potential mechanisms and modifications of dietary antioxidants on the associations between co-exposure to plastic additives and diabetes
Source: Nutr Diabetes. 2024 Sep 3;14:72. doi: 10.1038/s41387-024-00330-1 (PMC11372220; doi:10.1038/s41387-024-00330-1)

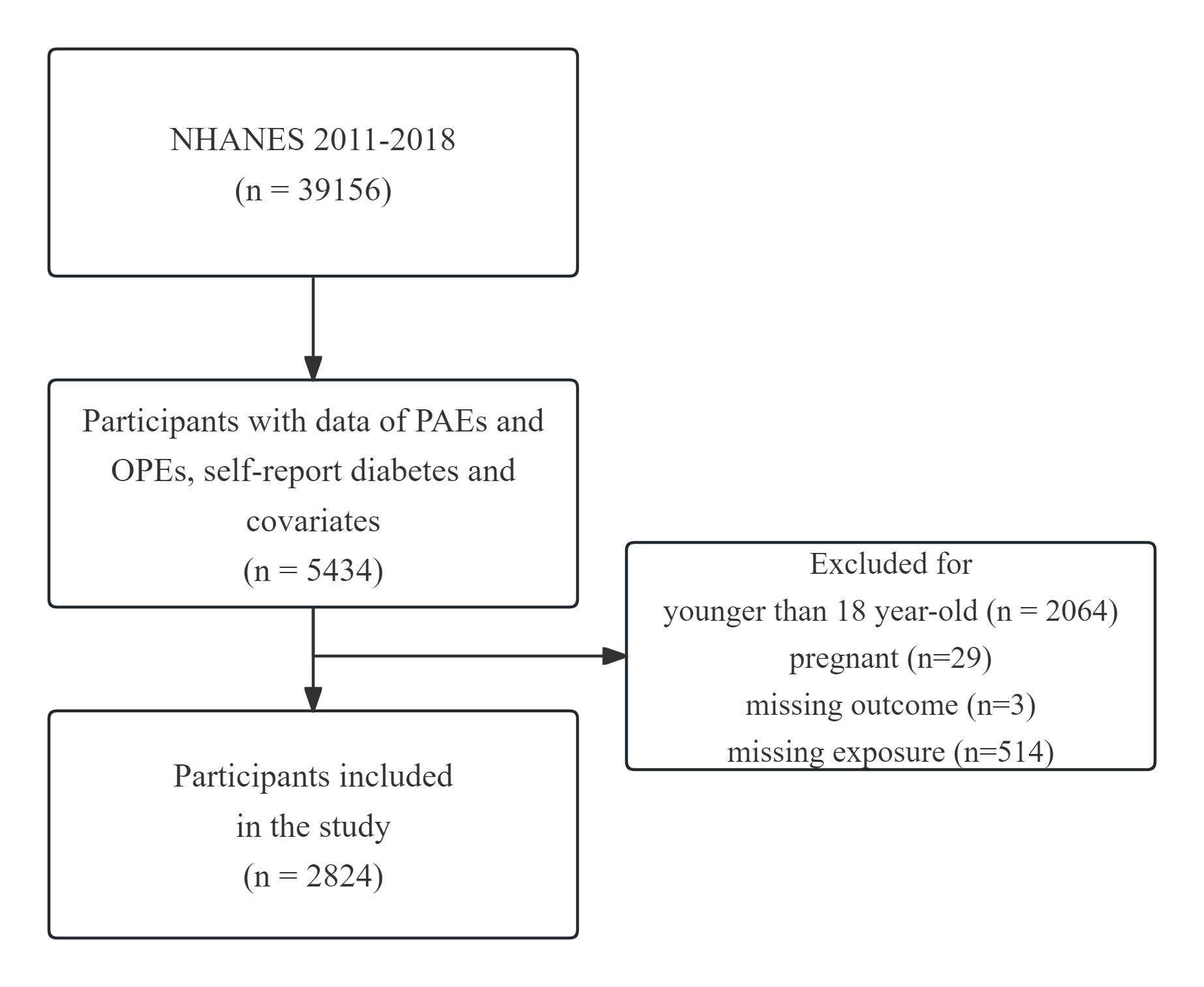

Supplement: Supplementary file 2 — figure s1 [file 41387_2024_330_MOESM2_ESM.png]

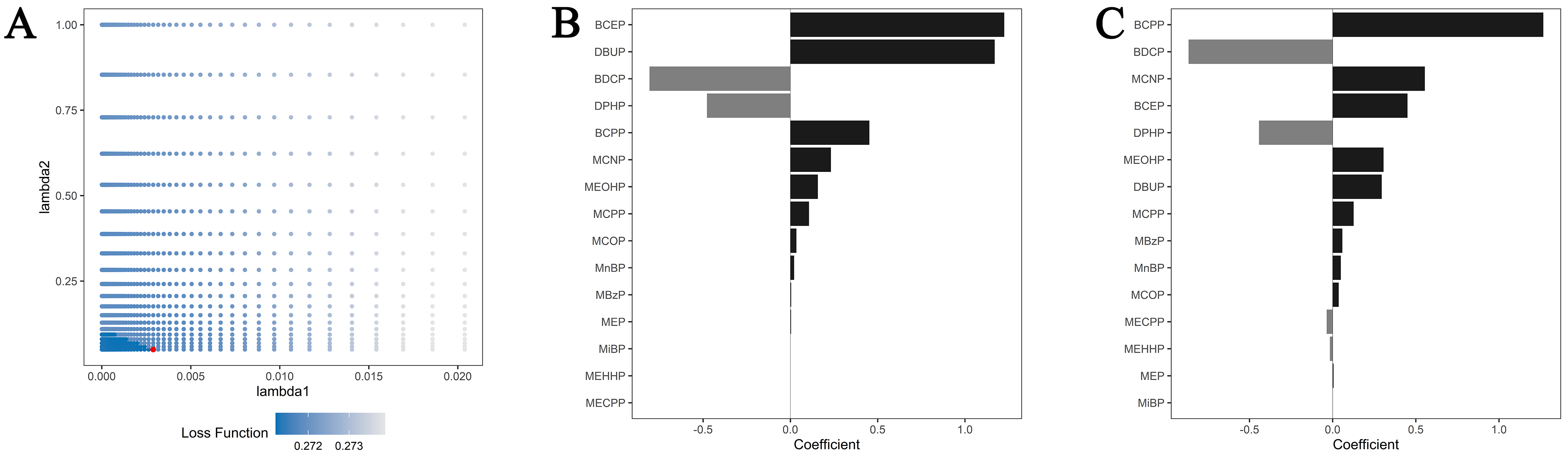

Supplement: Supplementary file 3 — figure s2 [file 41387_2024_330_MOESM3_ESM.tif]

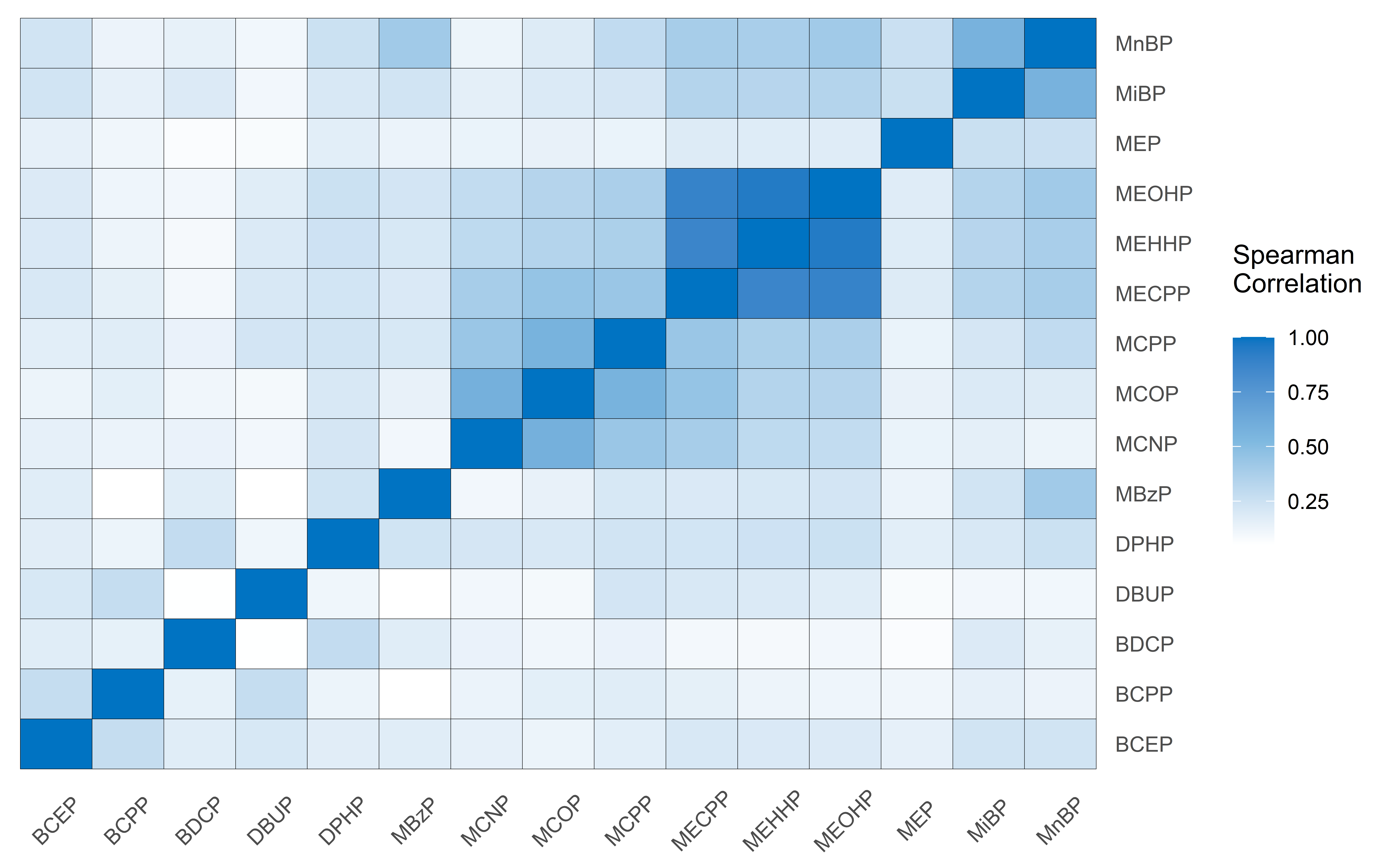

Supplement: Supplementary file 4 — figure s3 [file 41387_2024_330_MOESM4_ESM.png]

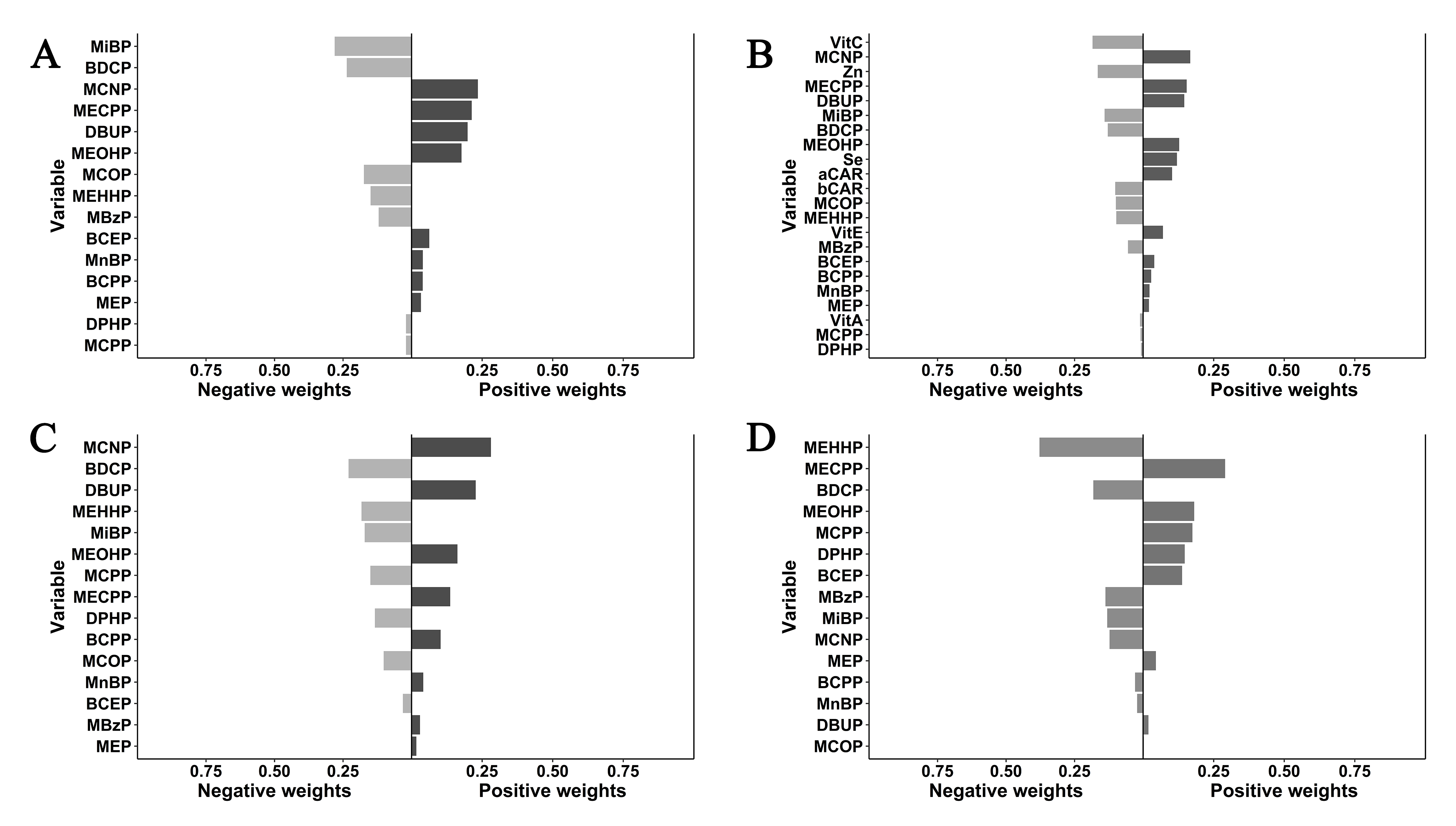

Supplement: Supplementary file 5 — figure s4 [file 41387_2024_330_MOESM5_ESM.tif]

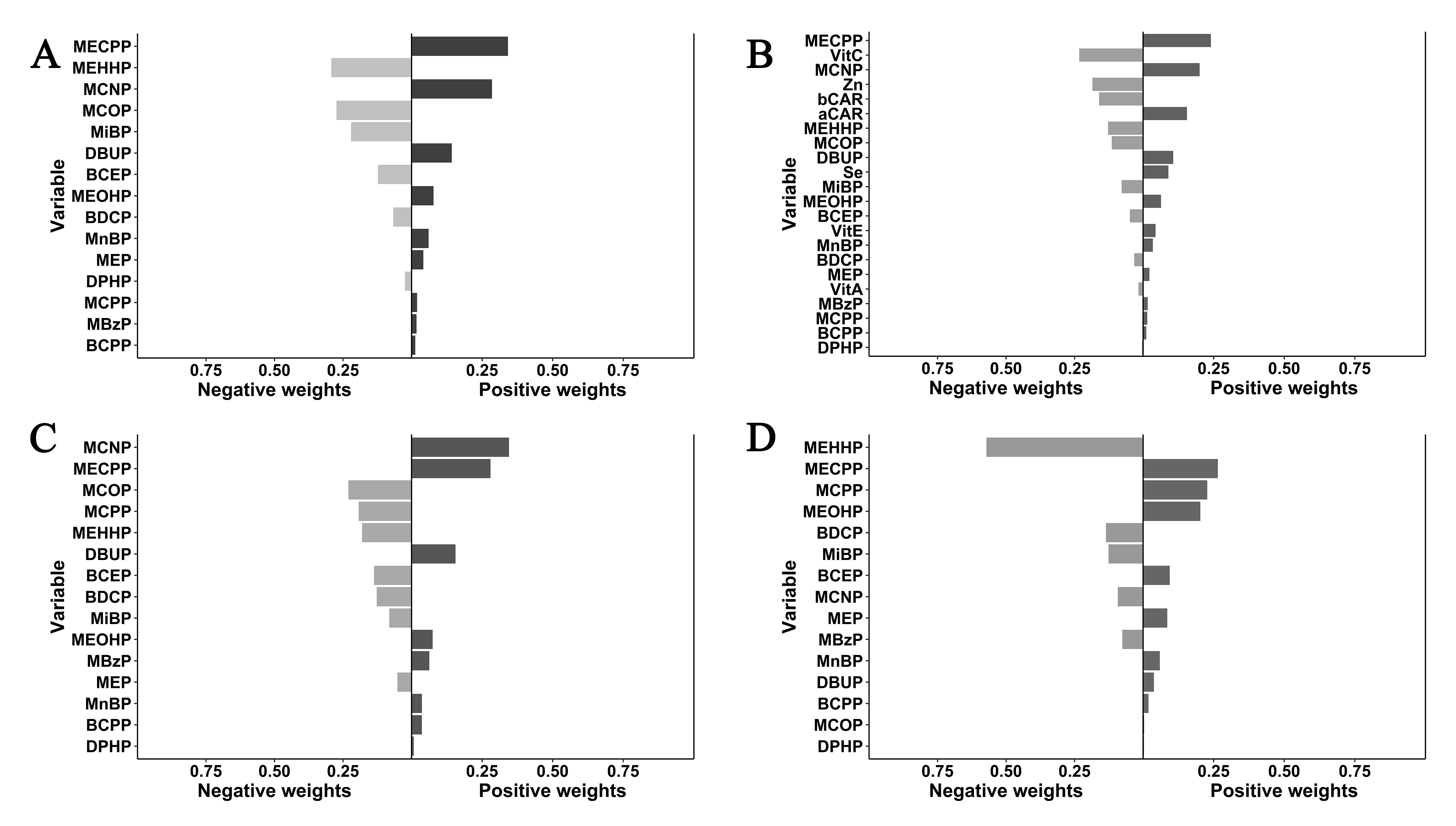

Supplement: Supplementary file 6 — figure s5 [file 41387_2024_330_MOESM6_ESM.tif]
